# p63 Promotes Cell Survival through Fatty Acid Synthase

**DOI:** 10.1371/journal.pone.0005877

**Published:** 2009-06-11

**Authors:** Venkata Sabbisetti, Arianna Di Napoli, Apryle Seeley, Angela M. Amato, Esther O'Regan, Musie Ghebremichael, Massimo Loda, Sabina Signoretti

**Affiliations:** 1 Department of Pathology, Brigham and Women's Hospital, Dana-Farber Cancer Institute, Harvard Medical School, Boston, Massachusetts, United States of America; 2 Department of Medical Oncology, Dana-Farber Cancer Institute, Harvard Medical School, Boston, Massachusetts, United States of America; 3 Department of Biostatistics and Computational Biology, Dana-Farber Cancer Institute, Harvard Medical School, Boston, Massachusetts, United States of America; 4 St. James's hospital, Dublin, Ireland; Roswell Park Cancer Institute, United States of America

## Abstract

There is increasing evidence that p63, and specifically ΔNp63, plays a central role in both development and tumorigenesis by promoting epithelial cell survival. However, few studies have addressed the molecular mechanisms through which such important function is exerted. Fatty acid synthase (FASN), a key enzyme that synthesizes long-chain fatty acids and is involved in both embryogenesis and cancer, has been recently proposed as a direct target of p53 family members, including p63 and p73. Here we show that knockdown of either total or ΔN-specific p63 isoforms in squamous cell carcinoma (SCC9) or immortalized prostate epithelial (iPrEC) cells caused a decrease in cell viability by inducing apoptosis without affecting the cell cycle. p63 silencing significantly reduced both the expression and the activity of FASN. Importantly, stable overexpression of either FASN or myristoylated AKT (myr-AKT) was able to partially rescue cells from cell death induced by p63 silencing. FASN induced AKT phosphorylation and a significant reduction in cell viability was observed when FASN-overexpressing SCC9 cells were treated with an AKT inhibitor after p63 knockdown, indicating that AKT plays a major role in FASN-mediated survival. Activated AKT did not cause any alteration in the FASN protein levels but induced its activity, suggesting that the rescue from apoptosis documented in the p63-silenced cells expressing myr-AKT cells may be partially mediated by FASN. Finally, we demonstrated that p63 and FASN expression are positively associated in clinical squamous cell carcinoma samples as well as in the developing prostate. Taken together, our findings demonstrate that FASN is a functionally relevant target of p63 and is required for mediating its pro-survival effects.

## Introduction

p63 (TP73L/TP63) is a homologue of the p53 tumor suppressor gene and represents the most ancient member of the p53 family [1]–[3]. Because of the presence of two promoters, p63 encodes two major classes of proteins: those containing a transactivating (TA) domain homologous to the one present in p53 (i.e. TAp63), and those that lack the TA domain (i.e. ΔNp63) [1], [2]. In addition, alternate splicing at the carboxy-terminal (C-terminal) generates at least three p63 variants (α, β, and γ) in each class.

Although p53 and p63 share high sequence and structural similarities, there are striking differences between their function and expression profiles. In physiological conditions, the expression of p63 is mostly restricted to the basal cell compartment of both stratified and glandular epithelia, with ΔNp63α being the predominant isoform [1], [4]–[8]. The analysis of p63-deficient mice unequivocally demonstrated that p63 plays a very critical role in the development of epithelial organs/tissues, including the epidermis and other squamous epithelia, salivary, lachrymal, mammary, and prostate glands and the bladder urothelium [4], [5], [9], [10]. Moreover, there is evidence that p63 is essential for the proliferative potential of stem cells both in the thymus epithelium and the epidermis [11]. In contrast to p53, p63 is rarely mutated in human tumors, and its role in cancer appears to be complex and needs to be further clarified [12]–[16]. Current data suggest that TA and ΔN isoforms might have opposite effects and support a role for ΔNp63 in promoting tumorigenesis.

ΔNp63 is highly expressed in up to 80% of primary head and neck squamous cell carcinomas (HNSCCs), as well as in malignancies of other squamous epithelia origin, including lung and esophageal cancers [15], [17]–[20]. Its overexpression is frequently associated with the amplification of the genomic locus at 3q27 that harbors the p63 gene [15], [17]. In addition, it has been proposed that aberrant p63 expression may be an early event in the pathogenesis of HNSCC, as extension of p63 expression is observed from normal basal cells into suprabasal levels in mucosal specimens displaying dysplasia [20].

The ectopic expression of ΔNp63 isoforms has been initially reported to inhibit p53 transactivation, leading to the early speculation that overexpression of ΔNp63 might simply be a mechanism to inactivate p53 [1], [21]. However, p63 inhibition has been shown to induce apoptosis by upregulating the pro-apoptotic Bcl-2 family genes *Puma* and *Noxa* through a mechanism that is independent of the p53 status of the cells but requires p73 [22]. Most importantly, subsequent studies have provided evidence that ΔNp63 also functions by directly activating the transcription of specific target genes [23]–[29]. For instance, it has been shown that p63 antagonizes apoptosis by regulating cellular adhesion in the basal cells of the mammary gland and other stratified epithelial tissues [30]. Nevertheless, the molecular mechanisms mediating p63 function both during development and tumorigenesis remain to be fully characterized.

Fatty acid synthase (FASN), a multifunctional enzyme that is essential for the endogenous synthesis of long-chain fatty acids from its precursors acetyl-CoA and malonyl-CoA [31], has been recently shown to be a direct target of ΔNp63α [32]. Given the increasing evidence that FASN is critical for cell survival and might act as a metabolic oncogene, we studied the functional relevance of FASN regulation by p63. We find that the pro-survival activity of p63 activity is significantly mediated by FASN in both transformed and immortalized epithelial cells.

## Materials and Methods

### Ethics Statement

All research involving human subjects has been conducted on anonimized, discarded tissues collected from patients during the course of their therapy. This research has been approved by the DF/HCC institutional review board. All animal work has been conducted according to relevant national and international guidelines.

### Cell lines and chemicals

The human squamous carcinoma cell line SCC9 was maintained in DMEM medium (Invitrogen Corporation, Carlsbad, CA) supplemented with 0.5 mM sodium pyruvate, 1.2 g/L sodium bicarbonate, 400 ng/ml hydrocortisone, and 10% FBS. SCC9-Tp63 and SCC9-Dp63 inducible clones, established as described below, were maintained in DMEM medium supplemented with 0.5 mM sodium pyruvate, 1.2 g/L sodium bicarbonate, 400 ng/ml hydrocortisone, 10% Tetracycline free FBS (BD Biosciences, San Jose, CA), 10 µg/ml blasticidin (Sigma-Aldrich, St. Louis, MO), and 300 µg/ml zeocin (Invitrogen Corporation, Carlsbad, CA). Immortalized prostate basaloid epithelial cells (iPrEC) (a gift from Dr. W.C.Hahn, Dana-Farber Cancer Institute, Boston, MA) were propagated in defined medium (PrEGM) (Lonza, Allendale, NJ), as recommended [33]. The FAS inhibitor C75 and PI3K inhibitor LY 294002 were purchased from Cayman chemicals, Ann Arbor, MI. The AKT specific inhibitor InSolution AKT inhibitor VIII, Isozyme-selective was purchased from EMD chemicals, Inc., Gibbstown, NJ.

### siRNA transfection

Tp63 siRNA (CGTATTCCACTGAACTGAA), targeting all p63 isoforms, Dp63 siRNA (GGACAGCAGCATTGATCAA), targeting ΔNp63 isoforms, and Scramble (CAGTCGCGTTTGCGACTGG) non-specific siRNA, were purchased from Dharmacon (Dharmacon Research Inc, Lafayette, CO). One day before transfection, SCC9 cells were seeded and incubated in DMEM supplemented with 0.5 mM sodium pyruvate, 1.2 g/L sodium bicarbonate, 400 ng/ml hydrocortisone and 10% FBS without antibiotics. The next day, cells at 40% confluency were transfected with p63 siRNA or Scramble siRNA using oligofectamine according to the manufacturer's instructions. For a 100 mm tissue culture dish, 2 ml of Opti-MEM1 reduced serum medium containing 40 nmol of siRNA and 40 µl of Oligofectamine (Invitrogen Corporation, Carlsbad, CA) were added to each plate. Studies involving smaller plates were scaled down according to the manufacturer's recommendations. Cells were harvested and processed further depending on the type of experiment.

### Generation of p63 shRNA inducible cell lines

The pBLOCK-iT inducible RNAi lentiviral expression system (Invitrogen Corporation, Carlsbad, CA) was used to establish Tetracycline inducible cells expressing shRNA for p63. First, SCC9 and iPrEC cells were infected with pLenti6/TR lentiviral expression construct according to the manufacturer's protocol. Stable transfectants were selected by culturing with Blasticidin (10 µg/ml for SCC9 and 3 µg/ml for iPrEC) (Sigma-Aldrich, St. Louis, MO). The clones with highest Tet repressor expression (SCC9 TR, iPrEC TR) were selected for further transfection with constructs expressing shRNA targeting all or ΔN-specific p63 isoforms. To this end, Tp63 and Dp63 sequences (see above) were inserted in pENTER/H1/TO entry vector. pENTER/H1/TO-Tp63 and pENTER/H1/TO-Dp63 constructs were then recombined with pLenti4/BLOCK-IT DEST Gateway vector to generate pLenti4/BLOCK-IT-Dp63 and pLenti4/BLOCK-IT-Tp63 shRNA expression constructs according to manufacturer's protocol. SCC9 TR and iPrEC TR clones were incubated with pLenti4/BLOCK-IT-Dp63 or pLenti4/BLOCK-IT-Tp63 shRNA viral supernatant and stable transfectants were screened with Zeocin (300 µg/ml for SCC9 and 50 µg/ml for iPrEC) (Invitrogen Corporation, Carlsbad, CA). The selected blasiticidin and zeocin resistant cells were screened for knockdown of p63 upon addition of tetracycline (2 µg/ml). Clones with efficient knockdown of p63 were selected and named SCC9-Tp63, SCC9-DP63, iPrEC-Tp63, and iPrEC-Dp63 depending on cell type and shRNA expression.

### Cell viability assay

Cell viability was assessed by using the 3-(4,5-dimethylthiazol-2-yl)-2,5 diphenyltetrazodium bromide (MTT) assay. MTT (5 mg/ml) was added to each well to a final concentration of 0.5 mg/ml and the plates were incubated for 3 h at 37°C. The formazan product was solubilized with 1 ml of dimethyl sulfoxide (DMSO) and the absorbance was measured at 590 nm using Perkin Elmer Victor^3^ multilabel counter (Perkin Elmer Life and Analytical Science, Shelton, CT).

### Flow cytometry assays

Cells were incubated with 10 µg/ml of BrdU for 2 h. Adherent cells were collected using trypsin-EDTA and floating cells were collected by centrifugation. The cells were combined, washed twice with PBS, fixed in 70% ethanol, and stored at −20°C. Fixed cells were permeabilized with 2M HCl, blocked with 0.5% heat inactivated serum for 1 h, and incubated in BrdU-FITC antibody (BD Biosciences, San Jose, CA) for 20 min at 4°C. After incubation, cells were washed with PBS and re-suspended in 300 µl of Propidium Iodide buffer (PBS, 0.1% Triton, 0.05 mg/ml RNase A, and 10 µg/ml Propidium Iodide) and incubated for 30 min at room temperature. The cells were then analyzed on a fluorescence-activated cell sorter (FACScan, BD Biosciences, San Jose, CA) using CellQuest software.

Annexin V staining was performed according to the manufactures protocol (BD Biosciences, San Jose, CA). Briefly, cells were washed twice with cold PBS and resuspended in 1× Binding buffer at a concentration of 1×10^6^ cells/ml. To 100 µl of this cells suspension, 5 µl of Annexin V antibody was added and incubated for 15 min at room temperature in the dark. After incubation, 400 µl of binding buffer was added to the tube and analyzed on a fluorescence-activated cell sorter (FACScan, BD Biosciences, San Jose, CA) using CellQuest software.

### Fatty acid synthesis assay

Fatty acid synthesis was measured by incorporation of [2-^14^C] acetate into lipids as described elsewhere [34], [35]. Briefly, cells were plated in 6 well plates at 3X10^5^ cells per dish and incubated overnight. The next day, cells were transfected with siRNAs, as described above. At several time periods after transfection, cells were incubated with 3 µCi of [2-^4^C] acetate per well for 4 hr. After incubation, cells were harvested and equal numbers of cells were suspended in 200 µl of PBS. Lipids were extracted with a 3∶1 methanol: chloroform mixture for 30 min with occasional shaking. After 30 min, an additional 250 µl of methanol and 300 µl of water were added. The samples were then centrifuged and the lower phase was counted for ^14^C using a Packard 1600CA Tri-Carb liquid scintillation counter (Packard Instrument Company, Downers Grove, IL). Each experiment was repeated in triplicates and the data was expressed as percent of the control.

### Western blot analysis

Equal amounts of protein (30 µg) were resolved electrophoretically and transferred to nitrocellulose membranes (Whatman GmbH, Dassel, Germany). After, blockading with 5% nonfat dry milk in Tris-buffered saline with 0.1% Tween 20, membranes were probed with the following primary antibodies: anti-p63 mouse monoclonal antibody (Santa-Cruz Biotechnology, Santa Cruz, CA), anti-FAS mouse monoclonal antibody (BD Biosciences, San Jose, CA), anti-phospho AKT rabbit polyclonal antibody (Cell Signaling Technology, Inc., Danvers, MA), anti-AKT rabbit polyclonal antibody (Cell Signaling Technology, Inc., Danvers, MA), anti-phosphoS6 rabbit polyclonal antibody (Cell Signaling Technology, Inc., Danvers, MA), and anti-cleaved caspase 3, rabbit monoclonal antibody (Cell Signaling Technology, Inc., Danvers, MA). Bound antibody was detected with HRP-conjugated goat anti-rabbit or goat anti-mouse (Thermo Fisher Scientific, Rockford, IL) and Immobilon™ Western chemiluminescent HRP substrate (Millipore Corporation, Temecula, CA).

### Real Time PCR studies

Total RNA was extracted using TRIzol reagent (Invitrogen Corporation, Carlsbad, CA) and cDNA was synthesized by reverse transcribing 1 µg of total mRNA using reverse transcriptase (Invitrogen Corporation, Carlsbad, CA), random hexamers (Invitrogen Corporation, Carlsbad, CA), and dNTP mixture according to the manufacturer's protocol. Real time PCR studies were performed using SYBR green (Applied Biosystems, Foster City, CA) on a Bio-Rad MyiQ™ single color Real-Time PCR detection system (Bio-Rad Laboratories, Hercules, CA). Each experiment was repeated in triplicates and data was analyzed using Gene Expression Macro™ software (Bio-Rad Laboratories, Hercules, CA).

### Squamous cell carcinoma tissue microarrays

De-identified formalin-fixed, paraffin-embedded head and neck squamous cell carcinoma (HNSCC) tissues were collected from 83 patients who were diagnosed and treated at the Brigham and Women's Hospital of Boston, and at the St. James Hospital, Dublin, Ireland. For each case, three core tissue biopsies (0.6 mm diameter) were taken from selected representative tumor areas and arrayed in three new recipient paraffin blocks. For 50% of the cases three additional tissue cores were taken from morphologically normal-appearing regions. Tumor characteristics are summarized in Table 1.

**Table 1:** Tumor characteristics of the HNSCC cases.

Tumor Characteristics	No. (%)
**Tumor grade**	
G1	8 (9.6%)
G2	38 (45.8%)
G3	37 (44.6%)
**pT stage**	
T1	16 (19.3%)
T2	31 (37.3%)
T3	14 (16.9%)
T4	22 (26.5%)
**pN stage**	
NX	8 (9.6%))
NO	35 (42.2%)
N1	11 (13.3%)
N2	26 (31.3%)
N3	3 (3.6%)
**pM stage**	
MX	70 (84.4%)
M0	9 (10.8%)
M1	4 (4.8%)

### Immunohistochemical and statistical analyses

Specimens were immunostained using the following antibodies: mouse anti-p63 (clone 4A4, dilution 1∶200, Santa-Cruz Biotechnology, Santa Cruz, CA) and rabbit anti-FASN (dilution 1∶200, Assay Designs, Ann Arbor, MI). Briefly, paraffin embedded tissue sections were deparaffinized and rehydrated and treated for antigen retrieval (0.01 M citrate buffer pH 6.0). This step was followed by incubation with 3% hydrogen peroxide to block endogenous peroxidase activity. The slides were then incubated with an optimal dilution of the primary antibodies for 60 minutes at room temperature. The reaction product was then developed using DAKO EnVision™+ System horseradish peroxidase detection kit (DAKO North America Inc, Carpinteria, CA) and counterstained with Hematoxylin. Double immunostaining was performed as previously described [36]. The double-immunostained slides were scanned using the ScanScope® slide scanning system (Aperio Technologies Inc., Vista, CA) and images analyzed by a Color Deconvolution Algorithm (Aperio Technologies Inc.).

Expression levels of each marker was evaluated by two pathologists (ADN and SS) blinded to clinical and laboratory data. p63 was scored by estimating the percentage of tumor cells displaying nuclear staining. Cytoplasmic expression of fatty acid synthase was scored as negative (no expression), weak expression (1+), moderate expression (2+), and strong (3+). The mean of all the three cores per tumor was used as a single value. A cutoff of 65% p63 positive cells was applied to separate high (>65%) and low (< or equal to 65%) p63 expressors. A FASN staining intensity ≥2 was applied to separate high and low FASN expressors. For frequency analysis in contingency tables, statistical analysis of associations between variables was performed by Fisher's exact test. Statistical significance was set at *P*≤0.05.

## Results

### Knockdown of either Total or ΔN specific p63 isoforms causes a decrease in cell viability

A squamous cell carcinoma cell line (SCC9) and immortalized prostate basaloid epithelial cells (iPrEC) were utilized to investigate the molecular pathways that mediate p63 function in epithelial cells.

**Figure 1:**
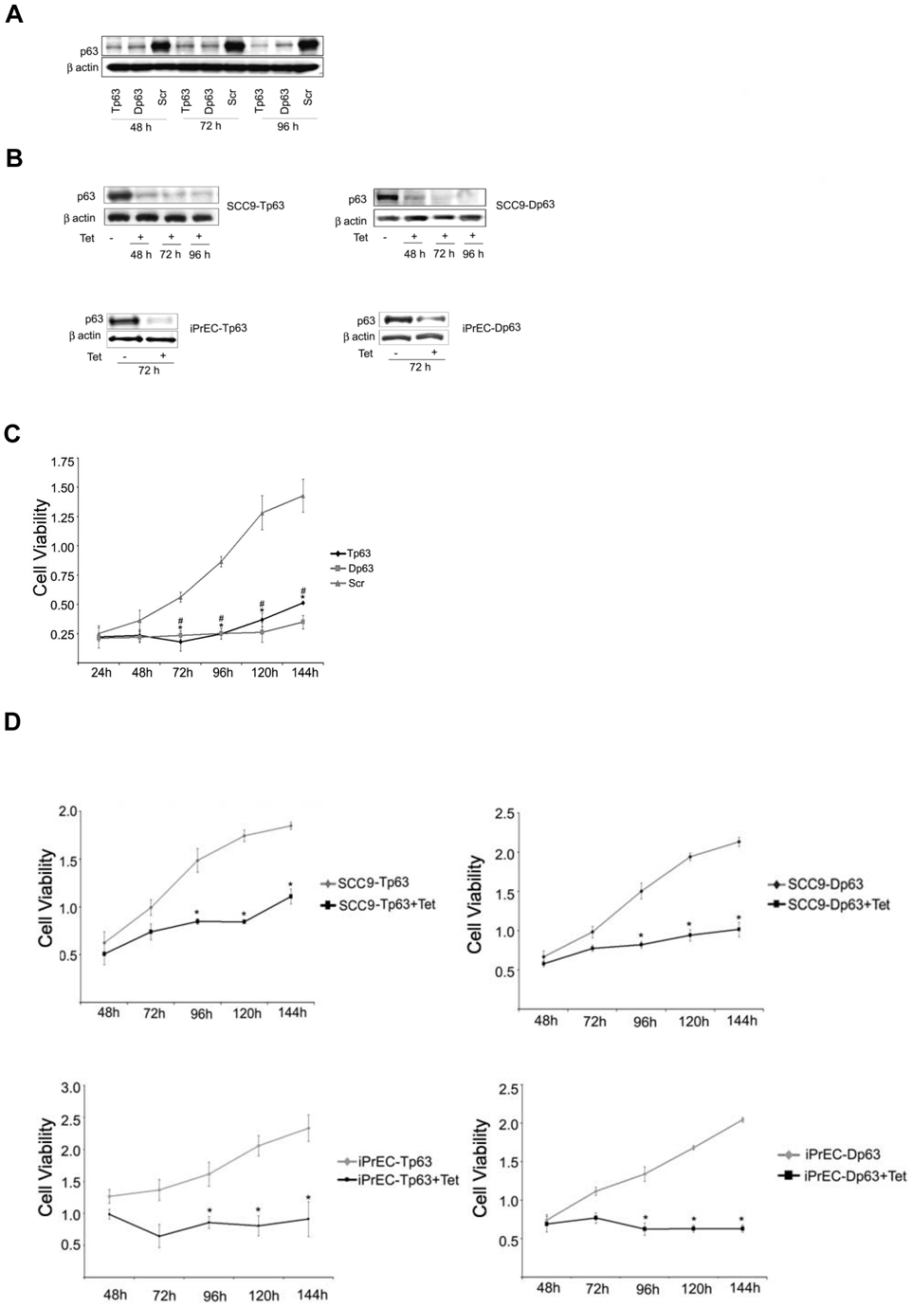
Silencing of either Total or ΔN specific p63 isoforms causes a decrease in cell viability. A. Immunoblot analysis of p63 in SCC9 cells transfected with Tp63, Dp63 or Scr siRNAs. B. Immunoblot analysis of p63 in tetracycline-inducible clones of SCC9 (SCC9-Tp63, SCC9-Dp63), and iPrEC (iPrEC-Tp63 and iPrEC-Dp63) with or without treatment with tetracycline. C–D. Cell viability curves. Quantification of cell viability measured by MTT assay in SCC9 cells transfected with Tp63, Dp63 or Scr siRNAs (C), in SCC9-Tp63 and SCC9-Dp63 cells with or without treatment with tetracycline (D, upper panel) and in iPrEC-Tp63 and iPrEC-Dp63 cells with or without treatment with tetracycline (D, lower panel). Data are shown as means +/− standard error and are representative of three independent experiments.

Silencing of p63 expression was performed in SCC9 cells utilizing siRNA oligos, targeting either all p63 isoforms (Tp63 siRNA), or ΔNp63 isoforms only (Dp63 siRNA). In addition, tightly regulated p63 silencing was achieved in both SCC9 and iPrEC cells by developing tetracycline-inducible clones expressing short hairpin RNA (shRNA) sequences for either all p63 isoforms (SCC9-Tp63 and iPrEC-Tp63) or ΔNp63 isoforms (SCC9-Dp63 and iPrEC-Dp63). Both RNAi methods led to a 60–80% decrease in p63 protein expression in the targeted cells (Figure 1A–B and data not shown). As expected, no changes in p63 protein levels were detected in cells transfected with control siRNA (Scr) or in clones that were not treated with tetracycline.

Downregulation of p63 expression in both SCC9 and iPrEC cells resulted in a significant decrease in cell viability. Specifically, knockdown of p63 in SCC9 cells caused a decrease in the number of viable cells as early as 48 hours after p63 siRNA transfection as compared to the scramble control (P = 0.02, two-tailed *t*-test) (Figure 1C). The effects on cell viability became even more evident at the later time points. By day 6 (144 h), there was a dramatic reduction in the viability of cells transfected with either Tp63, (P = 0.006, two-tailed *t*-test) or Dp63 (P = 0.0009, two-tailed *t*-test) siRNAs as compared to cells transfected with scramble siRNA. Similar results were observed upon p63 silencing in the inducible SCC9 and iPrEC clones (Figure 1D).

### Knockdown of either Total or ΔN specific p63 isoforms induces apoptosis without affecting the cell cycle

**Figure 2:**
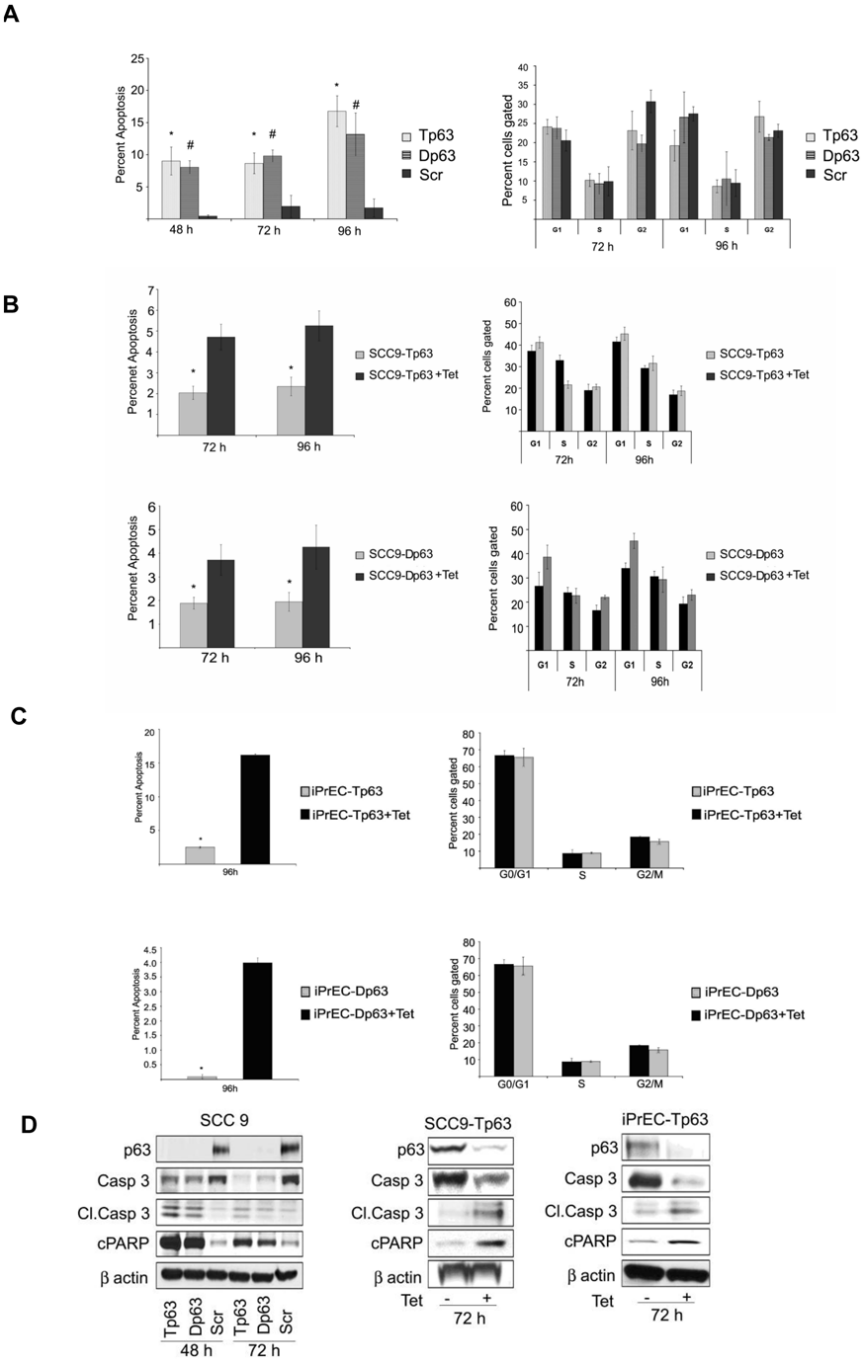
Silencing of either Total or ΔN specific p63 isoforms induces apoptosis with no significant changes in cell proliferation. A–C. Flow cyometric analysis of SCC9 cells transfected with Tp63, Dp63 or Scr siRNAs (A), SCC9-Tp63 and SCC9-Dp63 cells with or without treatment tetracycline (B) and iPrEC-Tp63 and iPrEC-Dp63 cells with or without treatment tetracycline (C). Left panels show quantification (percentage) of the sub G1 population measured by PI staining. Right panels show the fractions of cells in the various phases of the cell cycle, as determined by BrdU incorporation. Data are shown as means +/− standard error and are representative of three independent experiments. D. Immunoblot analysis of apoptosis-related proteins in SCC9 cells transfected with Tp63, Dp63 or Scr siRNAs (left panel), in SCC9-Tp63 cells with or without treatment with tetracycline (middle panel) and in iPrEC-Tp63 cells with or without treatment with tetracycline (right panel).

We next assessed whether the decrease in cell viability observed upon p63 downregulation was a result of apoptosis, cell cycle arrest, or both. We addressed this question by performing flow cytometric analysis in both SCC9 and iPrEC cell lines after p63 silencing. We found that p63 knockdown of either all p63 isoforms or ΔNp63 isoforms caused a significant increase in the sub G1 population of cells when compared to the control (P<0.05 at 48, 72, and 96 h, two-tailed *t*-test) (Figure 2A–2C, left panels). In line with these data, knockdown of p63 in both SCC9 and iPrEC cells resulted in an increase in the cleaved caspase 3 levels and a concomitant decrease in the expression total caspase 3. Accordingly, cleaved poly (ADP-ribose) polymerase (PARP) protein levels were increased after p63 knockdown (Figure 2D). These data provide evidence that p63 plays a role in the survival of both transformed and immortalized epithelial cells. The observation that Tp63 and Dp63 siRNAs produced similar results suggests that p63 anti-apoptotic function is mostly mediated by ΔNp63 isoforms.

In contrast to the effects on apoptosis, we did not observe any noticeable changes in the cell cycle distribution of the p63-silenced cells compared to their control (Figure 2A–C, right panels). These results indicate that p63 promotes cell survival without significantly affecting cell proliferation.

### p63 modulates Fatty Acid Synthase (FASN) levels and activity

It has recently been suggested that FASN is a conserved target of p53 family members, including p63 and p73 [32]. Since FASN has been shown to have anti-apoptotic activity, we investigated whether the pro-survival function of p63 is mediated by FASN.

**Figure 3:**
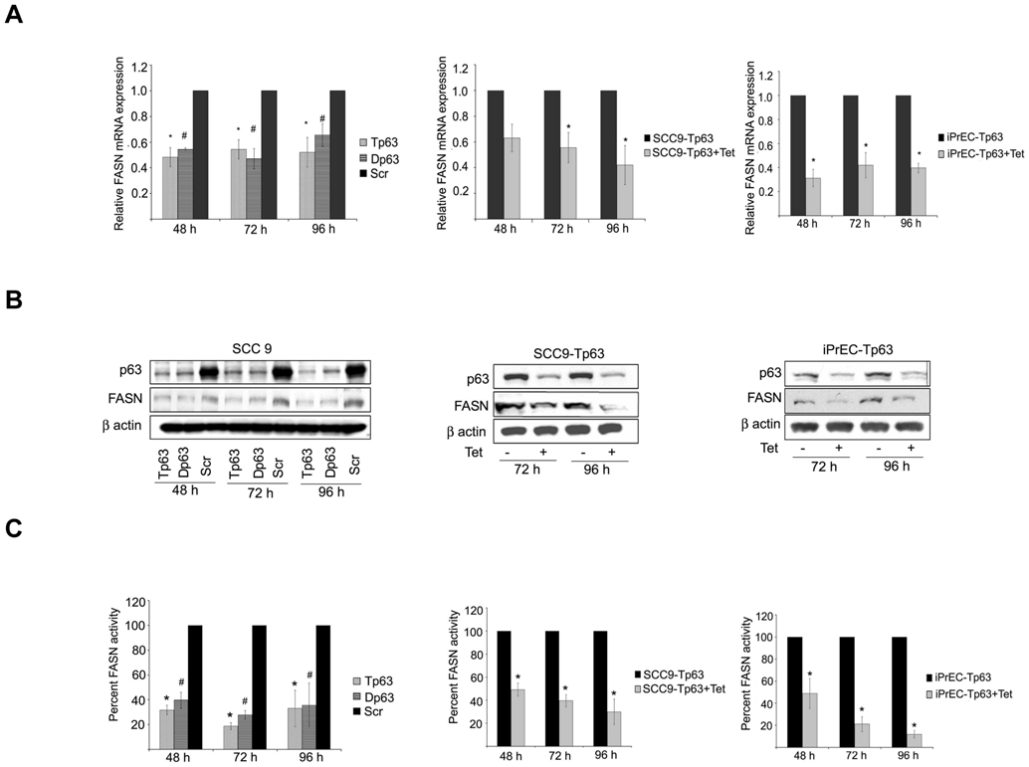
p63 silencing induces a decrease in FASN expression and enzymatic activity. A. Quantitation of FASN mRNA by real time RT-PCR in SCC9 cells transfected with Tp63 or Dp63 siRNA relative to cells transfected with Scr siRNA (left panel). Quantitation of FASN mRNA in SCC9-Tp63 and iPrEC-Tp63 cells treated with tetracycline relative to the correspondent untreated cells (middle and right panels, respectively). Data are shown as means +/− standard error and are representative of three independent experiments. B. Immunoblot analysis of FASN in SCC9 cells transfected with Tp63, Dp63 or Scr siRNAs (left panel), in SCC9-Tp63 cells with or without treatment with tetracycline (middle panel) and in iPrEC-Tp63 cells with or without treatment with tetracycline (right panel). C. Quantitation of C14 incorporation within cellular lipids in SCC9 cells transfected with Tp63 or Dp63 siRNA relative to cells transfected with Scr siRNA (left panel) and in SCC9-Tp63 and iPrEC-Tp63 cells treated with tetracycline relative to the correspondent untreated cells (middle and right panels, respectively). Data are shown as means +/− standard error and are representative of three independent experiments.

As first step, we assessed whether p63 regulates FASN expression by evaluating the effects p63 downregulation on FASN mRNA and protein levels. Knockdown of p63, with either Tp63 or Dp63 siRNAs decreased the mRNA expression of FASN by approximately 50% in the SCC9 cell line (Figure 3A, left and middle panels). In addition, we observed a similar decrease in FASN transcript levels after p63 knockdown in iPrEC cells (Figure 3A, right panel). Consistent with these results, we detected a significant decrease in FASN protein expression upon knockdown of p63 in both SCC9 and iPrEC cells (Figure 3B). These data confirm that p63 modulates FASN expression at both mRNA and protein levels in both transformed and immortalized epithelial cells.

In order to explore the functional consequences of FASN downregulation induced by p63 silencing, we investigated fatty acid synthesis in the p63-silenced cells by assessing C14 acetate incorporation into cellular lipids. In the SCC9 cell line, p63 knockdown resulted in a significant decrease in fatty acid synthesis compared to the control (p = 0.004, two-tailed *t*-test). Such decrease was observed by 48 hr after transfection with siRNAs and persisted up to the measured time point of 96 h (Figure 3C). Overlapping results were obtained in SCC9-Tp63 (p = 0.009, two-tailed t-test, 120 h) and iPrEC-Tp63 (p = 0.015, two-tailed *t*-test, 120 h) cells. Taken together, our data demonstrate that p63 silencing causes a significant reduction in both FASN levels and FASN enzymatic activity.

### p63 modulates AKT activation

**Figure 4:**
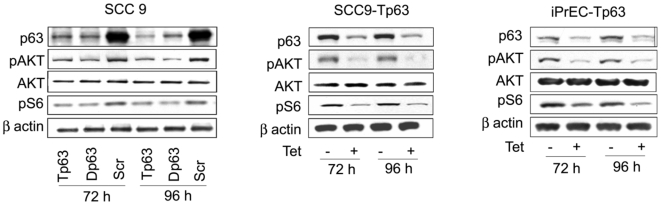
p63 silencing induces a decrease in AKT phosphorylation. Immunoblot analysis of total AKT, phospho-AKT (Ser 473), and phospho-S6 (Ser 235/236) in SCC9 cells transfected with Tp63, Dp63 or Scr siRNAs (left panel), in SCC9-Tp63 cells with or without treatment with tetracycline (middle panel) and in iPrEC-Tp63 cells with or without treatment with tetracycline (right panel).

Phosphatidylinositol-3′-kinase (PI-3′K)/protein kinase B (AKT) signaling has been extensively implicated in cell survival and there is some evidence that AKT might function both upstream and downstream of FASN [37]. Therefore, we also analyzed the role of p63 in modulating AKT activation in both transformed and immortalized epithelial cells. Silencing of p63 in both SCC9 and iPrEC cells induced a significant decrease in the phosphorylation of AKT without causing any changes in total AKT levels (Figure 4). In addition, we observed that p63 silencing resulted in a decrease in phosphorylation of ribosomal protein S6, which is a downstream effector of the PI-3K-AKT pathway.

### p63 pro-survival function are mediated by FASN and AKT

The observation that p63 regulates both AKT and FASN activity, suggests that signaling downstream of these molecules might be required for p63 anti-apoptotic function. We tested this hypothesis by investigating whether constitutive expression of FASN or activated-AKT is able to protect cells from the decrease in cell viability induced by p63 silencing. To this end, SCC9 and iPrEC cells were stably transfected with FASN, myristoylated AKT (Myr-AKT) or empty vector (EV). p63 silencing was achieved in the FASN overexpressing cells (FASN-SCC9 and FASN-iPrEC), Myr-AKT expressing cells (mAKT-SCC9 and mAKT-iPrEC) and control cells (EV-SCC9 and EV-iPrEC) by either transfection of siRNA or by generation of tetracycline-inducible clones expressing shRNA.

**Figure 5:**
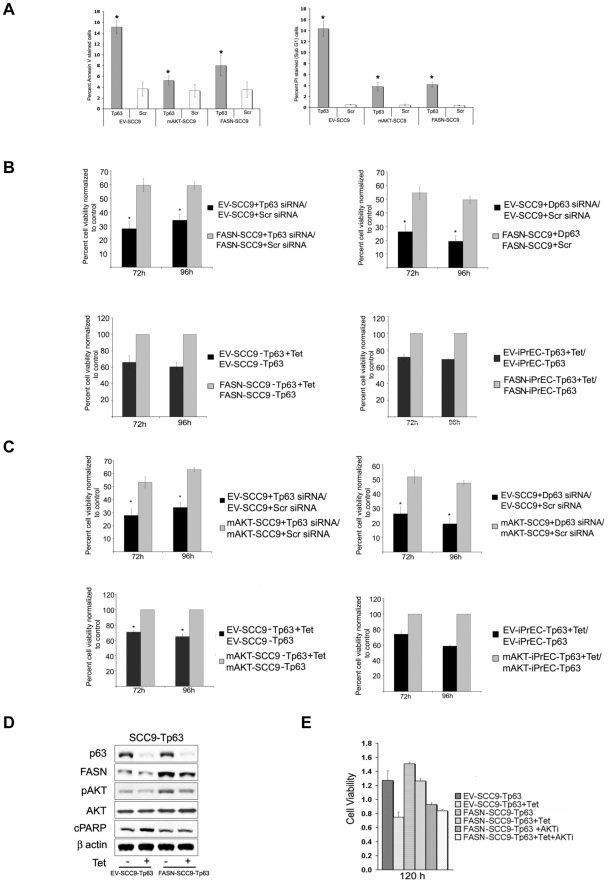
Increased FASN or AKT activity protects cells from apoptosis induced by p63 silencing. A. Quantitation of apoptosis by flow cytometry using Annexin V assay (left panel) or PI staining (right panel) in EV-SCC9, mAKT-SCC9, and FASN-SCC9 cells transfected with either Tp63 or Scr siRNAs. B. Effects of p63 silencing on the viability of SCC9 and iPrEC cells overexpressing either FASN or empty vector. Upper panels: Quantification of cell viability in EV-SCC9 and FASN-SCC9 cells transfected with either Tp63 (left panel) or Dp63 (right panel) relative to cells transfected with Scr siRNA. Lower left panel: Quantification of cell viability in EV-SCC9 and FASN-SCC9 cells carrying inducible Tp63 shRNA (EV-SCC9-Tp63 and FASN-SCC9-Tp63) treated with tetracycline relative to untreated cells. Lower right panel: Quantification of cell viability in EV-iPrEC and FASN- iPrEC cells carrying inducible Tp63 shRNA (EV-iPrEC-Tp63 and FASN- iPrEC-Tp63) treated with tetracycline relative to untreated cells. C. Effects of p63 silencing on the viability of SCC9 and iPrEC cells overexpressing either Myr-AKT or empty vector. Upper panels: Quantification of cell viability in EV-SCC9 and mAKT-SCC9 cells transfected with either Tp63 (left panel) or Dp63 (right panel) relative to cells transfected with Scr siRNA. Lower left panel: Quantification of cell viability in EV-SCC9 and mAKT-SCC9 cells carrying inducible Tp63 shRNA (EV-SCC9-Tp63 and FASN-SCC9-Tp63) treated with tetracycline relative to untreated cells. Lower right panel: Quantification of cell viability in EV-iPrEC and mAKT-iPrEC cells carrying inducible Tp63 shRNA (EV-iPrEC-Tp63 and mAKT-iPrEC-Tp63) treated with tetracycline relative to untreated cells. Data are shown as means +/− standard error and are representative of three independent experiments. D. Immunoblot analysis of p63, FASN, phospho-AKT and c-PARP in EV-SCC9-Tp63 and FASN-SCC9-Tp63 cells with or without treatment with tetracycline. E. Inhibition of AKT signaling significantly affects FASN-mediated rescue of cell death induced by p63 silencing. The graph shows the viability of EV-SCC9-Tp63 with or without treatment with tetracycline and of FASN-SCC9-Tp63 and FASN-SCC9-Tp63 cells with or without treatment with an AKT inhibitor and/or tetracycline. Data are shown as means +/− standard error and are representative of three independent experiments.

FASN and Myr-AKT overexpressing cells were monitored for p63 knockdown- induced cell death against cells expressing empty vector. Forced expression of FASN or activated AKT reduced the rate of apoptosis observed after p63 downregulation assessed by both annexin V and PI staining (Figure 5A). In addition, the effects of FASN and AKT activation on the viability of p63 silenced cells were assessed by comparing the decrease in number of viable cells caused by p63 downregulation in FASN and Myr-AKT overexpressing cells versus their respective controls. In accordance with the apoptosis data, constitutive expression of either FASN or Myr-AKT significantly, but not completely, rescued the decrease in cell viability caused by p63 down-regulation (Figure 5B, C). These results indicate that both FASN and AKT pathways play an important role in p63-mediated cell survival.

Cross talking between FASN and AKT has been suggested by various studies. There is some evidence that AKT signaling induces the expression of FASN and that inhibition of FASN activity results in downregulation of phospho-AKT [37]–[40]. In light of these previous observations, we investigated the cross talk between FASN and AKT in SCC9 cells.

Overexpression of FASN in SCC9 cells resulted in an increase in phosphorylation of AKT. Upon knockdown of p63, FASN-SCC9 cells showed a decrease in phospho-AKT expression to levels that were, however, still significantly higher than those detected in the EV-SCC9-Tp63 cells (Figure 5D and Figure S1B). These results suggest that the resistance to apoptosis conferred by FASN overexpression in p63-silenced cells could be at least in part due to activation of AKT signaling. In an attempt to better understand whether AKT plays a role in this process, we studied the effects of AKT inhibition in FASN overexpressing cells silenced for p63 expression. We observed a significant reduction in cell viability when FASN-SCC9 cells were treated with a specific AKT inhibitor (AKTi, 3 µM) after p63 knockdown (Figure 5E). Thus, AKT signaling appears to play a role in FASN-mediated survival.

Overexpression of myr-AKT or blockade of PI3K/AKT pathway (LY and AKT inhibitor) in the same cell line significantly modulated phospho-AKT levels but did not cause any alteration in the FASN protein levels, suggesting that AKT does not play a central role in the regulation of FASN expression in these cells (Figure S1). Surprisingly, however, FASN activity was significantly increased by overexpression of myr-AKT and decreased by AKT inhibition (Figure S2). This result raises the possibility of post-translational regulation of FASN by AKT and suggests that the rescue from apoptosis documented in the p63-silenced cells expressing myr-AKT may be partially mediated by FASN signaling. Moreover, this finding indicates that the reduction in viability caused by AKTi treatment in FASN-SCC9 cells could involve the inhibition of FASN activity. Of note, the effects of AKTi treatment on FASN-SCC9 cells appeared to be largely independent of p63 levels (i.e. similar reduction in viability was seen in p63-silenced and control FASN-SCC9 cells) (Figure 5E). Since ATKi inhibits both AKT and FASN activities, the observation that p63 levels have limited impact on the viability of FASN-SCC9 cells treated with ATKi is consistent with the hypothesis that AKT and FASN are important mediators of p63 pro-survival function.

### p63 and FASN expression are positively associated in human SCC tissues

**Figure 6:**
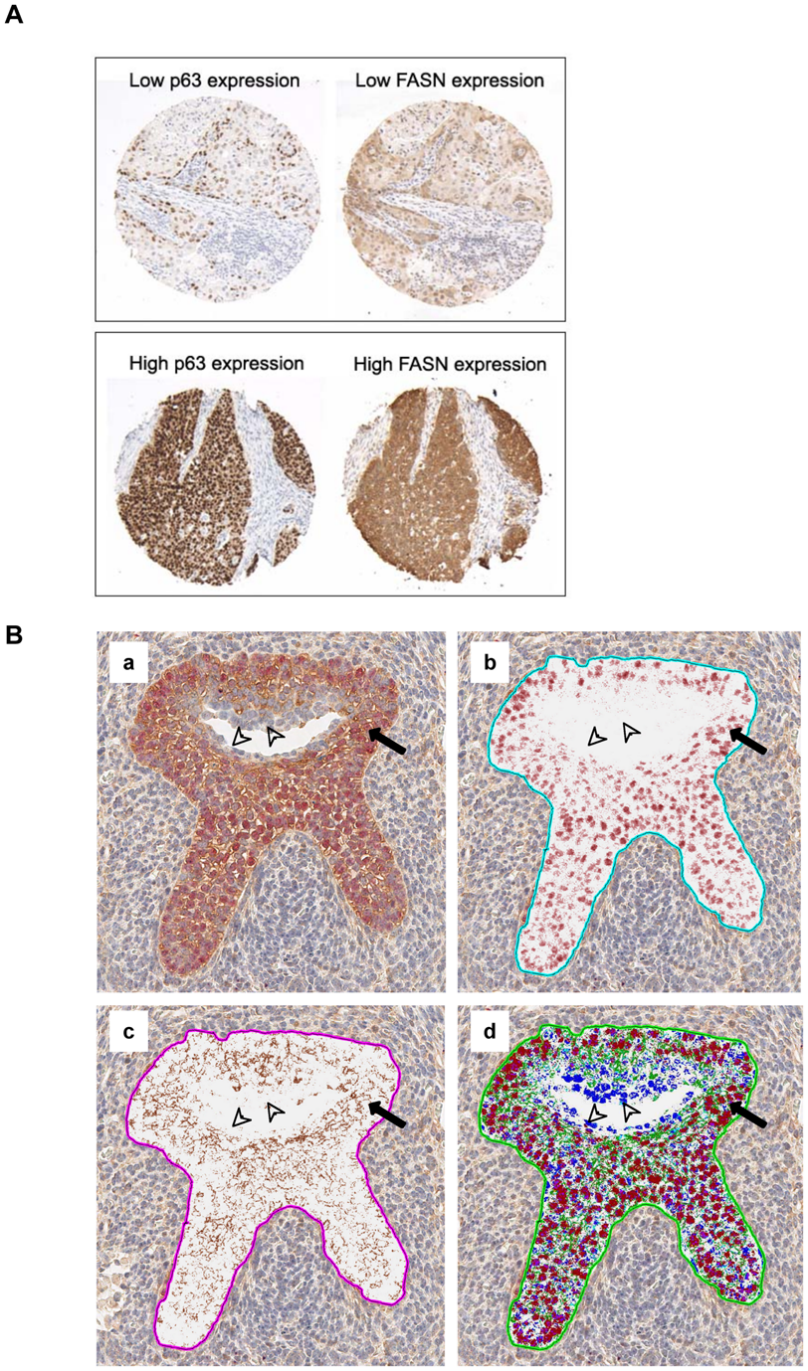
Co-localization of p63 and FASN *in vivo*. A. Immunohistochemical staining for FASN and p63 in TMAs containing cores from HNSCC tissues. Two representative cases are shown: one case displays both low p63 and low FASN expression (upper panel) and one case displays both high p63 and high FASN expression (lower panel). B. Co-localization of p63 and FASN in the UGS epithelium and prostatic buds. (a) A coronal section of the UGS from a wild-type E18.5 embryo was double immunostained for p63 (Fast Red) and FASN (DAB) and counterstained with hematoxylin. Deconvolved images of the DAB (b) and Fast Red (c) color channels as well a pseudo-colorized image (c) are also shown. In the pseudo-colorized image, the DAB channel (FASN) is displayed in green, the Fast Red channel (p63) is displayed in red and the hematoxylin channel is displayed in blue. Expression of both FASN and p63 proteins is observed in the vast majority of the cells. The cells lining the UGS lumen are consistently negative for both FASN and p63.

Our cell-based assays indicate that FASN is a functionally relevant target of p63 in SCC cells. If FASN were a main mediator of p63 function also in human tumors, the levels of the two proteins would be positively associated in SCC tissues. In order to test this hypothesis, we examined FASN and p63 protein expression by immunohistochemistry in a tissue microarray (TMA) containing cores from 83 cases of head and neck SCC (HNSCC). The characteristics of the tumors are described in Table 1. In the morphologically normal epithelium, FASN expression was weak and restricted to the lower layers. In line with previous reports, p63 expression was observed in both basal and parabasal epithelial cell layers (data not shown). Among the 83 tumors, FASN was detected in 95.2% of the cases and p63 in 98.8% of the cases. p63 protein expression was scored by estimating the percentage of positive nuclei in tumor cells. Cytoplasmic expression of FASN was scored as negative (no expression), weak expression (1+), moderate expression (2+), and strong (3+). Statistical analysis revealed a significant positive association (p<0.007, Fisher's exact test) between FASN and p63 expression levels (Table 2). Representative cases are shown in Figure 6A and Figure S3. Of note, FASN expression was more intense in the non-keratinizing poorly differentiated tumors with basaloid morphology, where p63 was found to be positive in almost 100% of tumor cells (Figure 6A, lower panel). No significant association was found between tumor grade and FASN or p63 expression levels (data not shown). These findings are in line with our *in vitro* data and further support a role for FASN in mediating pro-oncogenic p63 functions *in vivo*.

**Table 2:** Positive association of p63 and FASN expression in HNSCC cases.

FASN	p63		Total
	Low (≤65%)	High (>65%)	
**Low (<2)**	17 (44.74%)	21 (55.26%)	38
**High (≥2)**	7 (15.56%)	38 (84.44%)	45
**Total**	24	59	83

p<0.007, Fisher's exact test.

### p63 and FASN co-localize in the urogenital sinus (UGS) and developing prostate

We have previously shown that p63 is required for the development of prostate basal cells. The observation that the pro-survival role of p63 in the prostate basaloid iPrEC cells depends on FASN, prompted us to investigate a possible association between p63 and FASN expression in the developing prostate. To this end, transversal sections of the UGS from a mouse embryo at day E18.5 were double-immunostained for p63 and FASN. Co-localization of the two proteins was observed in the lower and intermediate layers of the UGS epithelium as well as in the prostate buds (Figure 6B). These results suggest that FASN may be an important mediator of p63 function during prostate development.

## Discussion

There is increasing evidence that p63, and specifically ΔNp63, plays a critical role in both development and tumorigenesis by promoting epithelial cell survival [4], [10], [11], [15], [27], [41]. However, very few studies have addressed the molecular mechanisms through which such important function might be exerted. In this respect, it has been suggested that ΔNp63 counteracts apoptosis by repressing the expression of pro-apoptotic genes and by activating a cell adhesion program [23], [24], [29], [30].

Here we find that p63 is indispensable for the survival of both iPrEC and SCC9 cells and provide substantial evidence that this process is mediated by the regulation of lipid metabolism, and specifically the function of FASN. Our findings show that p63 not only induces the expression of FASN at both the mRNA and the protein level but also enhances its enzymatic activity. Most importantly, stable overexpression of FASN is able to rescue cells from cell death induced by p63 downregulation.

FASN is a key enzyme that synthesizes long-chain fatty acids from acetyl-CoA and malonyl-CoA and is essential for embryonic development. Indeed, FASN knockout mice and most of the FASN heterozygous mice die in utero [42]. The up-regulation of FASN expression in numerous types of cancer and its association with poor outcome have long suggested its role in human malignancy [43]–[50]. In line with this hypothesis, inhibition of the FASN pathway by either FASN inhibitors including C75 or Cerulenin, or by silencing FASN expression with RNAi result in G1 arrest and/or induction of apoptosis in various cancers cells [51]–[56]. Moreover, recent findings from our group indicate that FASN functions as a metabolic oncogene in prostate cancer by inhibiting the intrinsic pathway of apoptosis [57]. Thus, the newly uncovered regulation of FASN activity by p63 establishes a novel functional link between this p53 family member and cellular metabolism. Moreover, it suggests that maintenance of fatty acid synthesis is one of the mechanisms through which p63 acts as a pro-survival molecule in both development and cancer.

D'Erchia et al. recently reported that FASN harbors two p53 responsive elements (REs) in its promoter site, and that TAp73α, and ΔNp63α, but not p53, TAp73β or TAp63α directly bind to these elements and modulate expression of FASN at the transcriptional level [32]. These data indicate that FASN is a direct target of ΔNp63 and are in agreement with our observation that p63 modulates both FASN transcript and protein levels. Moreover, in our experiments silencing of either total or ΔNp63 isoforms produced overlapping cell phenotypes, implying that ΔNp63 is the major player in controlling cell survival and FASN activity in epithelial cells.

The finding that p63 silencing decreases phosphorylation of AKT and of its downstream target S6 suggested that signaling through the PI3K/AKT pathway is involved in p63 function. This hypothesis was confirmed by the demonstration that ectopic expression of myr-AKT conferred protection from apoptosis induced by p63 loss. Our results are in line with those recently reported by Ogawa et al. demonstrating that activation of the AKT pathway is necessary for ΔNp63α protection against UV induced apoptosis [58].

A positive feedback loop between FASN and AKT activation has been proposed [37]. In agreement with previously published data [39], we observed that FASN sustains AKT activation, raising the possibility that AKT could function downstream of FASN in promoting cell survival. In line with this hypothesis, we found that blocking of the AKT pathway altered FASN-mediated rescue of cell death in the context of p63 silencing, Thus, AKT appears to be a critical mediator of FASN signaling, at least in our model system.

Activated AKT has been shown to increase the expression of FASN in several types of tumors including ovarian [37] and prostate cancers [59]. Accordingly, inhibition of the PI3K/AKT pathway with LY 294002 has been shown to reduce the expression of FASN [39]. In addition, there is evidence that the transcription activator SREBP-1c (sterol-regulatory-element-binding protein-1c), which is a known regulator of FASN expression, is modulated by both AKT and MAPK pathways [23], [37], [39], [40], [60], [61]. Surprisingly, we didn't observe any significant changes in FASN protein levels either by activating or blocking of the AKT pathway. Thus, the effects of the AKT pathway in regulating FASN levels might be cell type specific. In spite of this result, it is notable that forced expression of myr-AKT did significantly increase FASN activity, suggesting that additional and perhaps more general effects of the AKT pathway on post-transcriptional modification of FASN may be involved.

Overall, the findings that FASN induces AKT phosphorylation and is activated downstream of AKT highlight the presence of a positive-feedback loop between FASN and ATK signaling. Additional experiments are required to unravel the molecular mechanisms through which AKT signaling modulates FASN enzymatic activity without affecting its protein levels. It will also be important to determine whether p63 can induce AKT activation through mechanisms other than FASN signaling.

Overexpression of FASN as well as p63 has been previously demonstrated in squamous cell carcinomas from various organs [15], [18]–[20], [62]. In support of the hypothesis that p63 function is mediated by FASN, we observed that the expression of FASN and p63 were positively associated in human HNSCC tissues. Interestingly, FASN expression levels appeared to be highest in non-keratinizing poorly differentiated tumors with basaloid morphology that also expressed very high p63 levels. Moreover, in a number of well- to moderately-differentiated HNSCCs, p63 and FASN tended to co-localize in the least differentiated cells at the periphery of the lesion while more differentiated cells in the central part of the tumor nodules were mostly negative for the expression of both proteins. While these results are compatible with current data indicating that FASN is under the control of multiple oncogenic pathways [17], [60], [62]–[65], they seem to support a model in which p63 sustains FASN expression in the undifferentiated stem cell-like population of human HNSCC. As it has recently been suggested that p63 functions in maintaining the proliferative potential of epidermal stem cells [11], it will be critical to establish whether the same genetic program plays a role in cancer stem cells and whether FASN is involved.

FASN is essential for embryonic development and the FASN gene is broadly expressed in the mouse embryo [42]. By E9.5 FASN expression is evident throughout the epidermis and the strongest labeling is observed in the distal epidermis of the limb bud, including the apical ectodermal ridge (AER), a structure that is critical for limb development and also displays very high p63 levels [4]. The co-localization of FASN and p63 expression in various embryonic structures, including the UGS epithelium and developing prostate, supports a role for FASN as a critical mediator of p63 function during development.

## Supporting Information

Figure S1 Inhibition or activation of the AKT pathway does not alter FAS protein levels. A. SCC9 cells were treated for several time periods including 24 h, 48 h and 72 h with an AKT inhibitor (AKTi) and FASN protein expression was analyzed using western blotting. B. Overexpression of FASN and mAKT in SCC9-Tp63 clones. EV-SCC9-Tp63, mAKT-SCC9-Tp63, and FASN-SCC9-TP63 cells were treated with tetracycline for 72 h and western blotting was performed. Overexpression of mAKT didn't alter FAS protein levels. However, overexpression of FASN increased the levels of phospho-AKT.
(0.46 MB TIF)

Figure S2 Overexpression of myr-AKT or FASN increases FASN activity. FASN activity was assessed in SCC9, mAKT-SCC9, and FASN-SCC9 cells treated with an AKT inhibitor, AKTi (3 µM), or FASN inhibitor, C75 (10 µg/ml) or DMSO (control) for 4 h using. Both myr-AKT and FASN expression increased FASN activity compared to their controls. Blocking of either of these pathways with specific inhibitors decreased the activity of FASN.
(0.23 MB TIF)

Figure S3 Co-localization of p63 and FASN in HNSCC whole tissue section. Whole tissue section of a moderately differentiated HNSCC double stained for FASN (brown) and p63 (red). There is a gradient of expression of both p63 and FASN, decreasing from the periphery to the center of the lesion.
(5.10 MB TIF)
