# Expression of Concern: p63 Promotes Cell Survival through Fatty Acid Synthase

**DOI:** 10.1371/journal.pone.0219869

**Published:** 2019-07-11

**Authors:** The PLOS ONE Editors

After publication, concerns were raised about several western blot results reported in this article [1].

- The β-actin panel in Fig 4 for iPrEC-Tp63 cells appears similar to the β-actin panel in Fig 5D for SCC9-Tp63, with different aspect ratio. The original blots supporting these figures are no longer available, and so the authors are unable to resolve the questions regarding these control blots.
- It appears as though the same β-actin panels are presented in Fig 2D and in S1 Fig for SCC9 cells, although the p63 data and experimental conditions are different. The authors provided available blots in support of FASN, p-Akt, and β-actin results shown in S1B Fig (S1 File), which clarify that the incorrect β-actin blot was included for this experiment in the published figure. The original blots underlying Fig 2D and the p63 blot in S1B Fig are no longer available.
- p63 and β-actin data were reported multiple times in the article:
  ○ p63 in Fig 1A, Fig 3B, and Fig 4 for SCC9 cells
  ○ β-actin panels in Fig 1A, Fig 3B for SCC9 cells
  ○ p63 and β-actin panels for SCC9-Tp63 and iPREC-Tp63 cell lines in Fig 3B and Fig 4
  The authors commented that they believe the western blots in Figs 1A, 3B and 4 were obtained by analyzing proteins from the same experiments, and that the p63 data were presented multiple times in the article to demonstrate that the changes in FASN, p-Akt and pS6 levels were observed in cells in which they had also documented knockdown of p63 expression. The original SCC9 blots for p63 in Figs 1A, 3B and 4 and for β-actin in Figs 1A and 3B are provided in S2 File. The underlying blots are no longer available for the SCC9 β-actin blot shown in Fig 4, for the other SCC9 experiments shown in these figures, or for the iPREC-Tp63 or SCC9-Tp63 experiments.
- The same p63 data are presented in Figs 3B and 4 for the SCC9 cell line, although the β-actin data are different. The authors commented that the β-actin blot shown for SCC9 cells in Figs 1A and 3B also applies to the p63 experiment in Fig 4. The original blots are not available to clarify whether the β-actin blot shown in Fig 4 is the matched loading control for Akt, p-Akt, and p-S6 blots shown in this figure.
- The authors are unable to confirm whether the β-actin blots shown in the article’s figures are matched loading controls obtained using the same protein samples as in the corresponding experimental panels.

The *PLOS ONE* Editors post this Expression of Concern to notify readers of the unresolved issues pertaining to the control data reported in this article and the unavailability of primary data to support most of the western blot results.

## Supporting information

S1 File Original blot images underlying the β-actin, FASN, and p-Akt panels in S1 Fig. (PDF)

S2 File Original blot images underlying the p63 and corresponding β-actin experiments shown for SCC9 cells in Figs 1A, 3B and 4. (PDF)
