# Supplementary figures and images for: p63 Promotes Cell Survival through Fatty Acid Synthase
Source: PLoS One. 2009 Jun 11;4(6):e5877. doi: 10.1371/journal.pone.0005877 (PMC2691576; doi:10.1371/journal.pone.0005877)

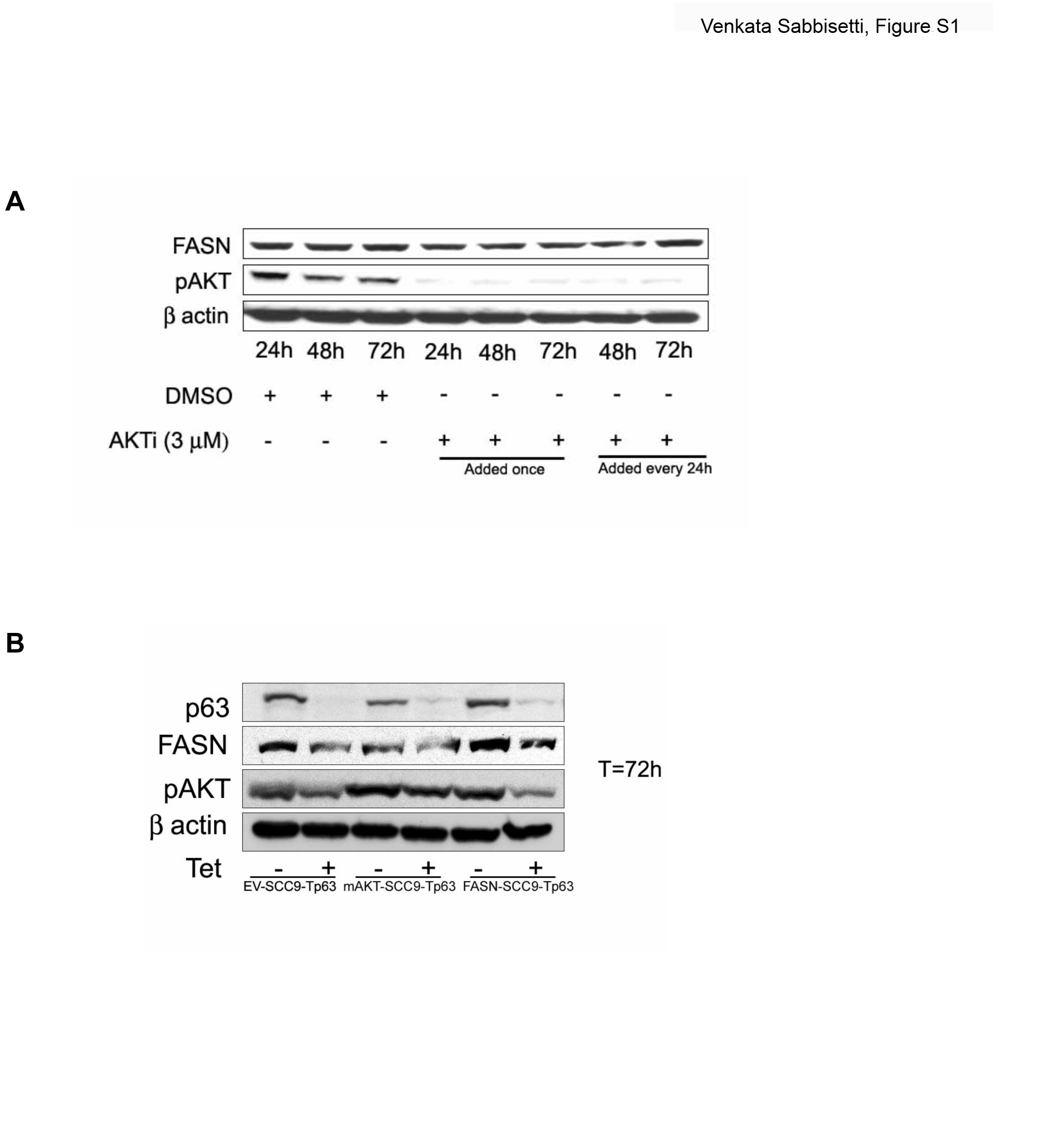

Supplement: Figure S1 — Inhibition or activation of the AKT pathway does not alter FAS protein levels. A. SCC9 cells were treated for several time periods including 24 h, 48 h and 72 h with an AKT inhibitor (AKTi) and FASN protein expression was analyzed using western blotting. B. Overexpression of FASN and mAKT in SCC9-Tp63 clones. EV-SCC9-Tp63, mAKT-SCC9-Tp63, and FASN-SCC9-TP63 cells were treated with tetracycline for 72 h and western blotting was performed. Overexpression of mAKT didn't alter FAS protein levels. However, overexpression of FASN increased the levels of phospho-AKT. (0.46 MB TIF) [file pone.0005877.s001.tif]

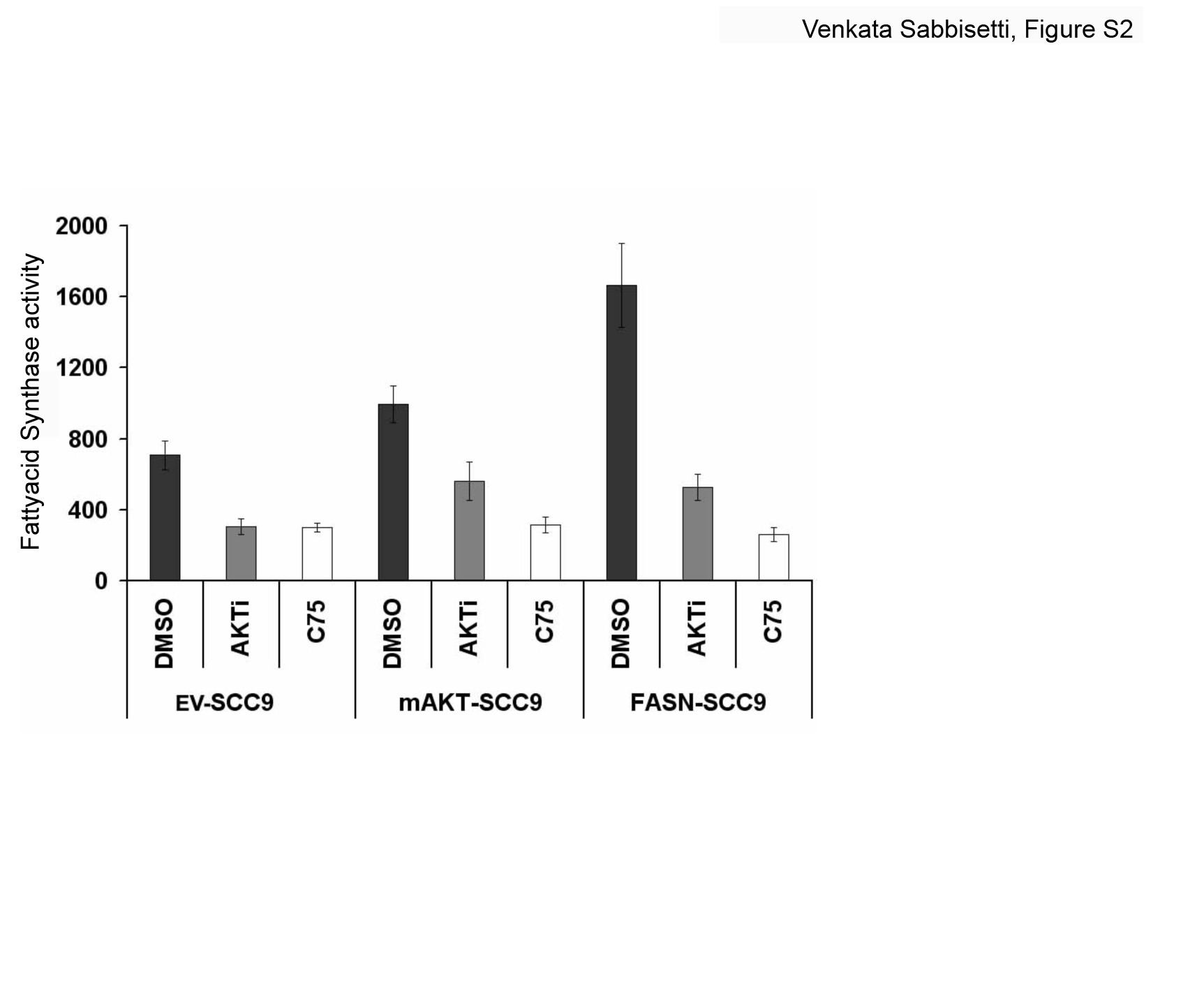

Supplement: Figure S2 — Overexpression of myr-AKT or FASN increases FASN activity. FASN activity was assessed in SCC9, mAKT-SCC9, and FASN-SCC9 cells treated with an AKT inhibitor, AKTi (3 µM), or FASN inhibitor, C75 (10 µg/ml) or DMSO (control) for 4 h using. Both myr-AKT and FASN expression increased FASN activity compared to their controls. Blocking of either of these pathways with specific inhibitors decreased the activity of FASN. (0.23 MB TIF) [file pone.0005877.s002.tif]

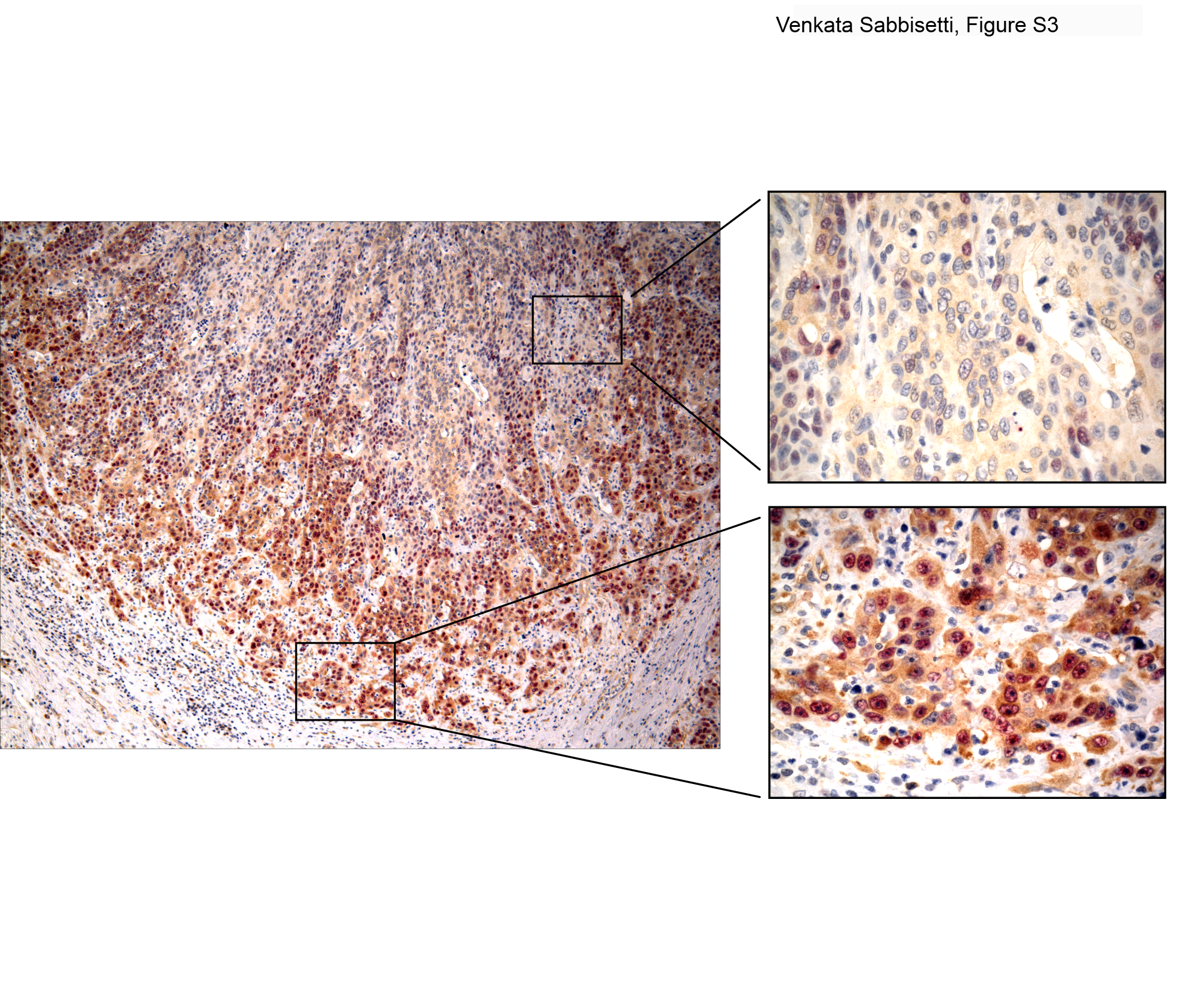

Supplement: Figure S3 — Co-localization of p63 and FASN in HNSCC whole tissue section. Whole tissue section of a moderately differentiated HNSCC double stained for FASN (brown) and p63 (red). There is a gradient of expression of both p63 and FASN, decreasing from the periphery to the center of the lesion. (5.10 MB TIF) [file pone.0005877.s003.tif]
