# Supplementary figures and images for: Expression of Concern: p63 Promotes Cell Survival through Fatty Acid Synthase
Source: PLoS One. 2019 Jul 11;14(7):e0219869. doi: 10.1371/journal.pone.0219869 (PMC6622553; doi:10.1371/journal.pone.0219869)

Fig.S1B

b-actin

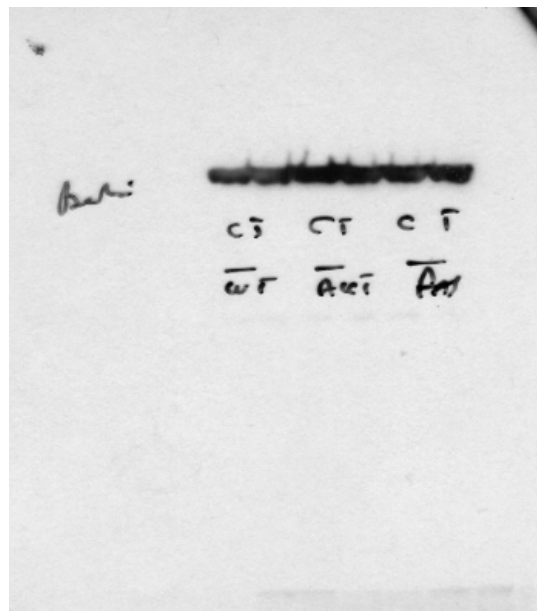

FASN

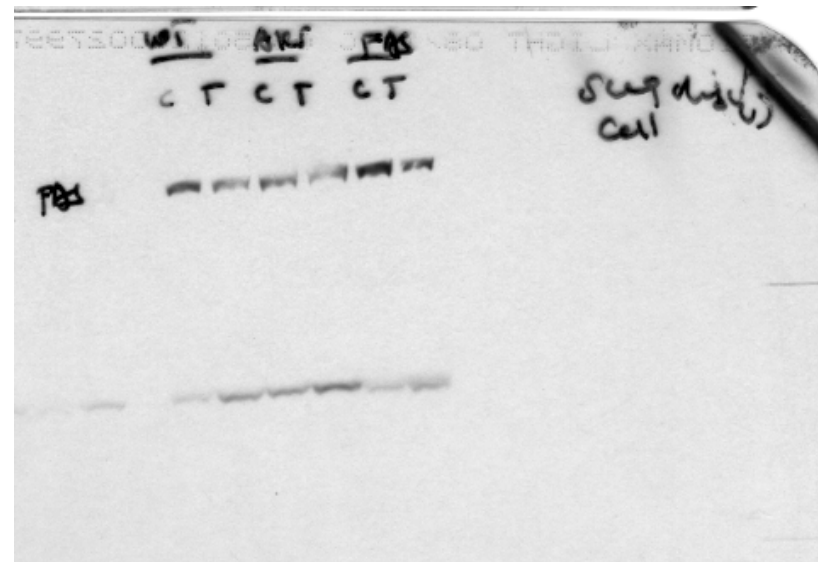

p-Akt

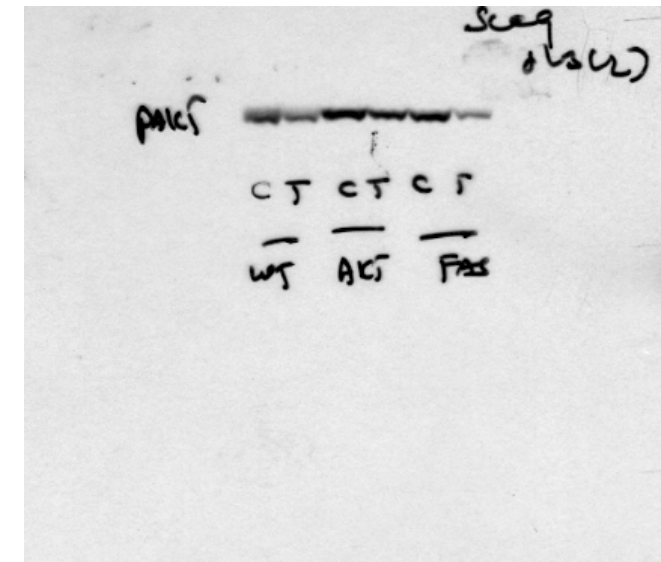

Supplement: S1 File — (PDF) [file pone.0219869.s001.pdf]

p63

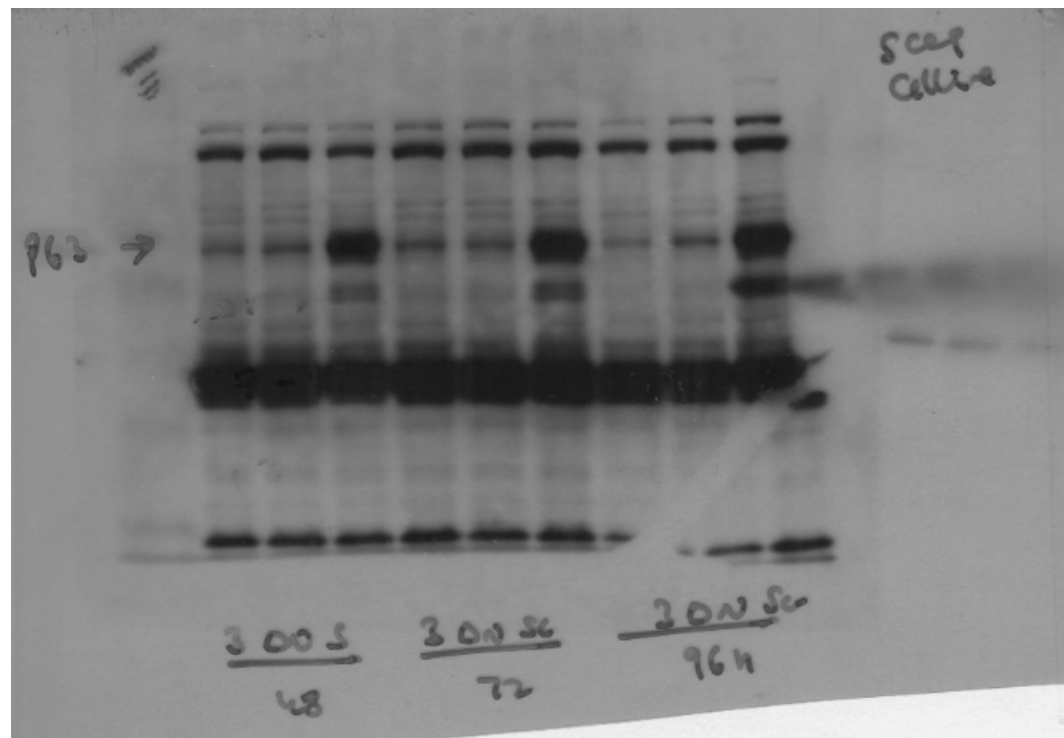

b-actin

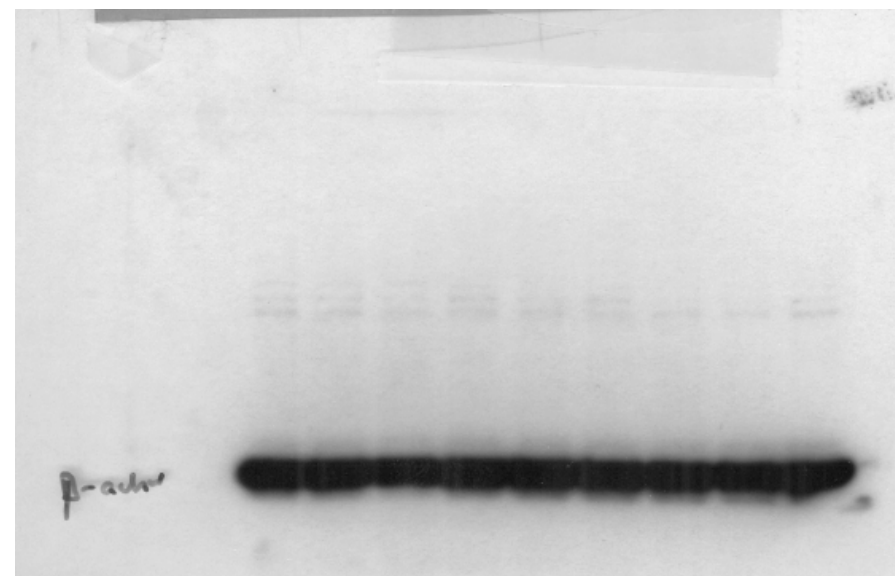

Supplement: S2 File — (PDF) [file pone.0219869.s002.pdf]
